# Impact of Real-Time Continuous Glucose Monitoring Use on Glucose Variability and Endothelial Function in Adolescents with Type 1 Diabetes: New Technology—New Possibility to Decrease Cardiovascular Risk?

**DOI:** 10.1155/2016/4385312

**Published:** 2015-11-16

**Authors:** Milena Jamiołkowska, Izabela Jamiołkowska, Włodzimierz Łuczyński, Joanna Tołwińska, Artur Bossowski, Barbara Głowińska Olszewska

**Affiliations:** Department of Pediatrics, Endocrinology, Diabetology with Cardiology Subdivision, Medical University of Białystok, Jana Kilinskiego 1, 15-089 Białystok, Poland

## Abstract

Children with type 1 diabetes (T1DM) are the high-risk group of accelerated atherosclerosis. Real-time continuous glucose monitoring (RT-CGM) provides possibilities for the detection of glycaemic variability, newly recognized cardiovascular risk factor. The aim of the study was to assess the usefulness of RT-CGM as an educational tool to find and reduce glycaemic variability in order to improve endothelial function in T1DM adolescents. Forty patients aged 14.6 years were recruited. The study was based on one-month CGM sensors use. Parameters of glycaemic variability were analyzed during first and last sensor use, together with brachial artery flow-mediated dilatation (FMD) to assess endothelial function. In the whole group, FMD improvement was found (10.9% to 16.6%, *p* < 0.005), together with decrease in all studied glycaemic variability parameters. In patients with HbA_1_c improvement compared to the group without HbA_1_c improvement, we found greater increase of FMD (12% to 19%, *p* < 0.005 versus 8.2% to 11.3%, *p* = 0.080) and greater improvement of glucose variability. RT-CGM can be considered as an additional tool that offers T1DM adolescents the quick reaction to decrease glycaemic variability in short time observation. Whether such approach might influence improvement in endothelial function and reduction of the risk of future cardiovascular disease remains to be elucidated.

## 1. Introduction

Type 1 diabetes mellitus (T1DM) is a well-established risk factor of accelerated cardiovascular disease, and, on the other hand, cardiovascular disease is the major cause of mortality in this group of patients. The relative risk for coronary heart mortality in T1DM patients is seven times higher than in matched counterparts without the disease and two times higher compared to risk of T2DM patients [1, 2]. Lately, it has been postulated that the main reason is not only chronic hyperglycaemia or other traditional risk factors, but frequent hypo- and hyperglycaemia episodes that accompany the disease daily course, and a new cardiovascular risk factor—excessive glycaemic variability—has been postulated. Blood glucose instability may contribute, perhaps even more than elevated HbA_1_c, to the development of diabetes complications [3, 4]. These extreme glucose fluctuations lead to endothelial dysfunction and accelerated atherosclerosis, independently of average glucose concentrations, probably in mechanism of oxidative stress occurrence [5, 6]. Alterations in endothelial function precede the development of morphological changes and contribute to atherosclerotic lesion development and progression [7]. It is now clear that vascular disease begins in childhood and progresses silently until complications, such as stroke or myocardial infarction, later appear. T1DM in childhood is recognized as a high-risk factor for premature atherosclerosis [8–10].

Appreciation of the role of the vascular endothelium throughout the atherosclerotic disease process has led to the development of a range of invasive and noninvasive techniques which permit evaluation of different aspects of its function [11]. At present, several noninvasive imaging techniques used in children offer an opportunity to study the relationship of surrogate markers to the atherosclerosis development. The use of these techniques may help to identify high-risk individuals in preclinical phase who may benefit from active therapy to prevent clinical disease. Endothelial function, that is, the vasodilator response to increased blood flow (flow-mediated vasodilatation, FMD), can now be accomplished using high-resolution ultrasound. FMD is an early endothelial dysfunction marker, used to detect minimal endothelial changes in children and adolescents [12].

T1DM poses a challenge to the goal of maintaining adequate glucose variability for years. From the 1990s, a new device has been introduced to the market to support patients in achieving this goal. Real-time continuous glucose monitoring system (RT-CGM) continuously measures the glucose level in interstitial fluid of subcutaneous tissue [13]. It provides the ability to track blood glucose trends and, thanks to warning alarms, prevents dramatic glucose variability before these changes become problematic. The potential clinical benefit of this technology as a tool to assist with optimization of glucose control has been demonstrated in several recent clinical studies, reviewed in [14].

The studies demonstrate that strict T1DM control helps not only in HbA_1_c and glycaemic variability reduction, but also in diminishing oxidative stress level and dyslipidemia. Both of these are predominant in children and adolescents with T1DM, playing a key role in endothelial dysfunction and leading to early atherosclerosis development [15–17]. Some recent data indicate that even up to thirty percent of pediatric T1DM patients have impaired endothelial function, independently associated with A_1_c and related to endothelial nitric oxide synthase T(-786)C polymorphism to some extent [18].

Noteworthily, there are no studies so far assessing possible influence of the RT-CGM device use on the cardiovascular system neither in T1DM adults nor in children. We hypothesized that use of RT-CGM via reduction of glycaemic variability might influence improvement of endothelial function. Therefore, we aimed firstly to assess brachial artery flow-mediated dilatation before and after short-term use of real-time continuous glucose monitoring in adolescents with T1DM. Secondly, we aimed to define a possible group of young patients who would benefit most from the use of this method.

## 2. Subjects and Methods

### 2.1. Patients

The entire study involved a total of forty adolescents (21 girls) at the age of 11–18 years—mean 14.6 ± 2.1, with the diagnosis of T1DM—who had been treated by continuous subcutaneous insulin infusion (CSII). Mean disease duration was 7.4 ± 3.7 years and mean HbA_1_c level at the baseline was 9.35 ± 1.53%. Primary eligibility requirements were diabetes duration above 1 year, exogenous insulin requirement at least 0.5 j/kg/day, and insulin pump therapy for at least 0.5 years. Patients were excluded from the study if they presented other diseases that could affect the outcome (additional autoimmune disease, hypertension, hyperlipidemia, obesity, and early microangiopathy) as well as having education difficulties and presenting a lack of cooperation. We enrolled patients who have never used real-time continuous glucose monitoring system before but expressed their willingness to participate in the study. All of the patients used either a sensor augmented insulin pump (Paradigm 722, Medtronic MiniMed, Northridge, CA) or Guardian RT (Medtronic, Northridge, CA) device when the original pump (Paradigm 715, Medtronic or AccuCheck Spirit, Roche) did not have a sensor option. We used “enlite” sensors (Medtronic) in all patients.  Table 1 presents the basic characteristic of the study group.

### 2.2. Study Design

The study was based on one-month continuous glucose sensors use combined with technical training and proper education of the patients and their caregivers concerning insulin therapy, diet, and physical activity. There was also the written instruction of operating system given to all of the patients. Children and their parents were able to contact the diabetology team if any problem occurred. Every patient was supplied with 4-5 sensors, changed every 6-7 days, to provide the 4-week trial. First sensor was inserted by medical staff. After the first and last sensor usage, the obtained data were uploaded to the computer using the CareLink Pro (Medtronic). Clinical issues were analyzed and discussed together with diabetologist. During the study, patients were told to live normal life adjusting the insulin therapy based on sensor records, confirmed with SMBG measurements, by themselves or prior telephone consultations. They also calibrated sensors 4 times a day using the personal glucometer. Next, we performed the analysis of computer uploaded glucose variability parameters that included mean blood glucose, standard deviation (SD) for the mean glucose, and area under the curve (AUC) for glucose level >140 mg/dL and <70 mg/dL, minimum and maximum glucose levels.

### 2.3. Laboratory Analyses

The level of glycated hemoglobin (HbA_1_c) was determined by high-pressure liquid chromatography (HPLC) at the baseline and 3 months after the study. Depending on the initial value of HbA_1_c, the group was divided into well-controlled group (with HbA_1_c <7.5%, mean 7.25 ± 0.19%) and poorly controlled group (with HbA_1_c >7.5%, mean 9.88 ± 1.22%). Decrease of HbA_1_c by at least 0.5% after 3-month follow-up was the criterion to divide the study group into improved group versus not improved group.

### 2.4. Ultrasound Measurements

The ultrasound measurement procedure was conducted between 8.00 and 10.00 AM after a fasting period of 8–12 hours. Examinations of the brachial and carotid arteries were performed with Hewlett Packard Sonos 4500 apparatus, using a 7.5 MHz linear transducer. Ultrasound examination of the right brachial arteries was performed in longitudinal sections 2–10 cm above the elbow, according to guidelines [19].

The principle is to induce vasodilatation in the proximal (brachial) artery by postischemic (forearm) enhanced flow. All lumen diameter measurements were scanned at end diastole by use of the R-wave of the electrocardiogram. First scans were taken at rest and second scans during reactive hyperemia. Increased flow was induced by deflating a pneumatic tourniquet placed on the right forearm, inflated to the pressure about 50 mmHg above the patient's resting systolic blood pressure for 4.5 min. The postischemic scan was performed 45–120 seconds after cuff deflation. Flow-mediated dilatation (FMD) was derived from the percentage change of the brachial artery diameter after ischemia of the forearm from baseline. Nitric oxide-dependent FMD of the brachial artery was assessed at baseline (before RT-CGM sensor placement) and after one month of sensors usage.

The study was approved by the Bioethics Committee, Medical University of Białystok, Poland. Caregivers and children were informed about the purpose and nature of the study. The caregivers gave a written consent, whereas the children expressed a spoken consent before examination.

### 2.5. Statistical Analysis

The statistical analysis was performed using the Statistica 9.0 software (Krakow, StatSoft). To determine the differences between the study groups for variables with normal distributions, the Student *t*-test was applied. The Student *t*-test for paired variables was used to compare the variables within the respective groups at baseline, after one month (glycaemic variability parameters), and after 3 months (HbA_1_c values). Since in this study the variables satisfied the conditions of normal distribution, no other tests were applied. The correlations between studied variables were assessed using Pearson correlation. All the results are presented as the mean ± standard deviation (SD). Differences at *p* < 0.05 were considered statistically significant.

## 3. Results

### 3.1. Metabolic Control and Glucose Variability

Our analysis of metabolic control in the study group was based on HbA_1_c level, which has significantly decreased in entire study group after three months from 9.35 ± 1.5% to 8.81 ± 1.8% (*p* < 0.001). Significant HbA_1_c improvement, which we considered as at least 0.5%, 3 months after the study was observed in 68% of the patients (27 children). In the group with improvement, HbA_1_c decreased from 9.03 ± 1.4% to 8.04 ± 1.2% (*p* < 0.001). The group with no improvement in HbA_1_c showed significantly worse metabolic control already at the beginning of the study: 10.03 ± 1.7% versus 9.03 ± 1.4% (*p* = 0.054 compared to group with improvement), and that difference has grown much more after three-month follow-up: 10.42 ± 1.6 versus 8.04 ± 1.2% (*p* < 0.001) (Table 2). In the group of children with optimal metabolic control (HbA_1_c <7.5%), mean HbA_1_c level decreased from 7.25 ± 0.2% to 6.73 ± 0.2% (*p* = 0.006), and in the group with poor metabolic control (HbA_1_c ≥ 7.5%), mean HbA_1_c level after 3-month follow-up decreased from 9.88 ± 1.2% to 9.33 ± 1.16% (*p* = 0.003) (Table 3).

Glucose variability parameters during the last week sensor have improved compared to results of first week sensor use in the entire study group. We noticed decrease of mean glycaemia, SD of mean glycaemia, and maximum glucose values and AUC for hyperglycaemia, while minimum glucose values increased and AUC for hypoglycaemia did not change (Table 2). The significance of that improvement was related to the HbA_1_c level changes. Mean glucose decreased significantly in all the patients after a monthly usage of RT-CGM, but some other parameters have changed significantly only in group with metabolic improvement. SD for mean glucose in these patients decreased from 60.74 mg/dL during the first sensor usage to 51.67 mg/dL during the last sensor usage (*p* = 0.010). Area under the curve (AUC) >140 mg/dL decreased from 41.23 using the first sensor to 21.22 using the last sensor (*p* < 0.001). Maximal glucose level changed from 344.37 mg/dL to 317.41 mg/dL (*p* = 0.004). The positive effect of RT-CGM use lost some statistical significances in group without HbA_1_c improvement (Table 2).

When we compared children with initial optimal and poor glycaemic control, we noticed that the largest favourable changes were found in group with HbA_1_c >7.5%. All glycaemic variability parameters, apart from AUC <70 mg/dL that remained stable, achieved improvement at level *p* < 0.001. In group with initial optimal HbA_1_c, only minimum glucose level achieved significant improvement: 42.75 mg/dL versus 48 mg/dL, *p* = 0.045. Noteworthily, however, the glucose variability parameters were better at the beginning of the study and at the end as well in the group with initial optimal glycaemic control when compared appropriately with first and last sensor use in poor control group (Table 3).

### 3.2. Endothelial Function

**Figure 1:**
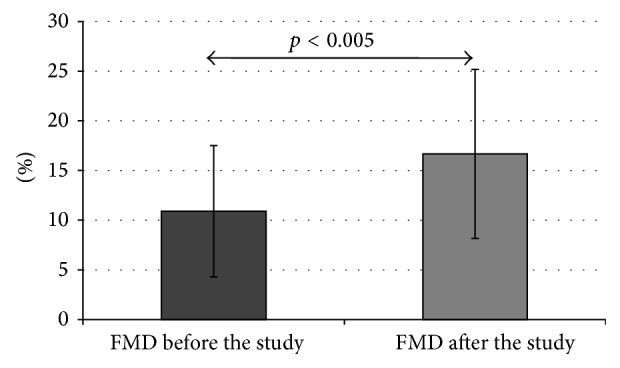
FMD in the whole study group before and after the study (data presented as mean ± SD).

**Figure 2:**
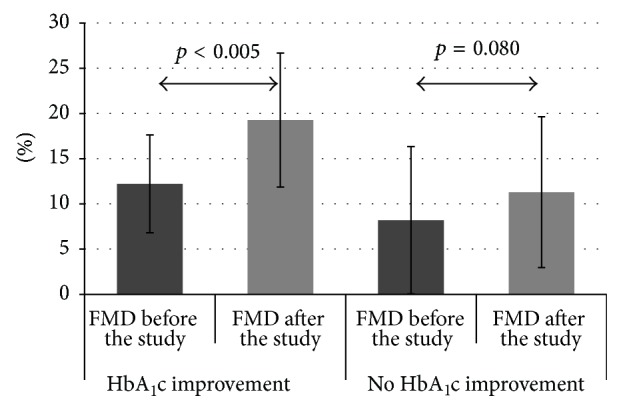
FMD changes depending on HbA_1_c improvement in follow-up (data presented as mean ± SD).

In the whole study group, an increase of FMD of the brachial artery was observed (from 10.90 ± 6.6% to 16.67 ± 8.5%, *p* < 0.005) (Figure 1). This result depended on metabolic control improvement. A much greater increase of FMD was found in the patients with HbA_1_c improvement (from 12.22 ± 5.4% to 19.27 ± 7.4%, *p* < 0.005) compared to the group without HbA_1_c improvement (from 8.18 ± 8.2% to 11.29 ± 8.4%, *p* = 0.080) (Figure 2). As we have subdivided our patients into two groups depending on the initial HbA_1_c level (>7.5% and <7.5%), we have found a greater increase of mean brachial artery FMD in a group with initial HbA_1_c below 7.5% (from 16.25 ± 4.3% to 27.81 ± 6.5%, *p* < 0.005) than in a group with initial HbA_1_c above 7.5% (from 9.57 ± 6.4% to 13.89 ± 6.4%, *p* < 0.005).

### 3.3. Correlation Analysis

We found that FMD before the study significantly correlated inversely with HbA_1_c before the study (*r* = −0.6, *p* < 0.001), mean glucose before the study (*r* = −0.40, *p* = 0.009), and AUC >140 mg/dL before the study (*r* = −0.35, *p* = 0.026). FMD after the study significantly correlated inversely with HbA_1_c after the study (*r* = −0.78, *p* < 0.001), mean glucose after the study (*r* = −0.37, *p* = 0.016), mean glucose from the whole study (*r* = −0.44, *p* = 0.004), and AUC >140 mg/dL for the whole study (*r* = −0.34, *p* = 0.27). No associations were observed between subclinical marker of atherosclerosis and total cholesterol, LDL-cholesterol, and triglycerides or between disease duration and onset either.

## 4. Discussion

The present study is the first to our knowledge to show that use of real-time CGM can be helpful in reduction of glycaemic variability in order to improve endothelial function in young T1DM patients without any symptoms of clinically present cardiovascular disease. Among the whole study group, we have observed significant endothelial function improvement, mainly in those patients who have also improved their HbA_1_c level. In those patients, the initiation of RT-CGM caused both a rapid reduction of glucose variability and improvement in vascular function.

Glucose variability affects quality of life and can contribute to pathogenesis of diabetic complications [4, 20]. Continuous subcutaneous insulin infusion (CSII) and multiple daily insulin injections (MDI), established therapies for type 1 diabetes, are thought to prevent hyperglycaemia and deleterious glucose fluctuations. Patients using insulin pumps present with lower glycaemic variability, better glycaemic control, and treatment satisfaction compared with those using MDI [21, 22]. RT-CGM can be considered as one step further in achieving a safe way to target near normoglycaemia. Although there is still a debate as to whether RT-CGM can improve glycaemic control and improve quality of life, increasing amount of data of observational and RCT studies in the pediatric age groups found improvement in metabolic control with use of this device. Most of the RCT studies, including pediatric T1DM, have demonstrated that the frequency of the CGM use was significantly associated with the effect of lowering HbA_1_c levels. Real-time CGM has been shown to lead to sustained reduction in MAGE and hypoglycaemia; nevertheless, benefits from sensor augmented pumps relate to sensor use frequency [13, 14]. Our results are in agreement with these studies, as we observed significant improvement in HbA_1_c in almost 70% of patients and significant improvement in glucose variability parameters.

Recently, the close relationship between increased glucose variability and endothelial dysfunction has been demonstrated in several studies in both type 1 and type 2 diabetic patients [5, 23, 24]. Noteworthily, in the study of obese adults with or without metabolic syndrome and type 2 diabetes, results of CGM revealed the close connection between glycaemic variability and endothelial function. Interestingly, it appeared that glycaemic variability may be elevated even in nondiabetic, despite being obese, subjects and is also independently correlated with endothelial function [24]. Furthermore, one of Australian studies demonstrated that children with T1DM following continuous subcutaneous insulin infusion (CSII) initiation have early improvements in vascular function, blood pressure, and metabolic control associated with reduced glucose variability [23]. In our study, all of our patients have already used CSII. Moreover, we have gone a step further and used real-time glucose monitoring to give the patients the possibility of positive feedback and immediate reaction. In the discussed studies, patients were using CGM blindly, and the results were analyzed retrospectively, whereas in our study patients were given a possibility for dynamic modulation of the displayed glycaemic variability. They could react immediately by changing the insulin dosage, diet, physical activity, and lifestyle. In the abovementioned study, the effects were unfortunately not sustained after twelve months, and FMD and glucose variability returned to baseline levels, with deterioration of metabolic control. Poor adherence to CSII-related tasks, such as insulin bolusing for meals, is frequently seen in adolescents [25]. In our study, we have discovered that using real-time CGM may help to improve not only glucose variability parameters, but endothelial function as well, in patients already treated with CSII.

In some contrast to our results remains the study of Peña et al., where only hypoglycaemia, but not glucose variability, during continuous glucose monitoring related to impaired vascular endothelial function in children with T1DM [26]. However, this was not interventional study, like our study was. In our group, we found that AUC for hypoglycaemia and minimal glycaemia value in the whole study group did not change significantly after one-month glucose sensor use. Interestingly, however, in the group with initial optimal glycemic control, where the highest improvement in endothelial function was observed, we noticed higher minimal glucose value during last week of sensor use compared to the first sensor use and insignificantly decreased AUC for hypoglycaemia.

It has been speculated that oxidative stress is the link between glucose variability and vascular dysfunction. Both in vitro studies and animal models have demonstrated evidence of vascular inflammation and endothelial cell apoptosis following fluctuations in blood glucose levels, via the production of reactive oxygen species [27, 28]. Oscillating blood glucose may have much worse effect on endothelial function and oxidative stress than stable hyperglycaemia in diabetic as well as nondiabetic subjects.

CGM allows constant glucose variability control. Still, the gold-standard method to measure glucose variability in research and clinical practice was not established. Using just glycated hemoglobin (HbA_1_c) as a glycaemic variability parameter has a number of limitations: it presents average glucose levels from the last three months, while the research shows that avoiding hypo- and hyperglycaemia episodes may be even more important. In some patients, it is even possible to reduce glycaemic instability without HbA_1_c improvement. The superiority of glycaemic variability was presented in recent studies, which recommend that clinicians should focus first on limiting glucose variability, before attempting to reduce median blood glucose, that is, HbA_1_c [29]. Standard deviation (SD), an index of the dispersion of data around mean blood glucose, was by design viewed as the simplest approach for the evaluation of glucose variability, beyond the simple determination of mean blood glucose. A clear consensus on the gold-standard method to measure glucose variability in clinical practice and research is still lacking, although a number of indicators have been proposed [30]. In our study, we have chosen generally accepted methods of the quality of blood glucose control and variability. These include the area under the curve (AUC) and the percentage of time inside, above, and below the blood glucose target. We have intentionally avoided the use of newly introduced parameters, such as continuous overlapping net glycaemic action (CONGA) or mean amplitude of glycaemic excursions (MAGE) due to reports showing the limitations of those methods and to the fact that those have not gained widespread use in clinical practice [24, 31].

The impact on endothelial function seems to be more effective in the patients who present better adherence. Our study has confirmed the correlation of FMD increase with metabolic control improvement. In our study, we named responders to CGM these patients who were able to improve the HbA_1_c level at least by 0.5% after three months of follow-up. Almost 70% of our adolescents achieved this result, and what is more, responders achieved greater improvement in endothelial function as FMD increased in this group significantly more than in nonresponders. Patients who improved their metabolic control presented greater decrease of parameters of glucose variability—the average glucose, SD for the mean glucose, AUC >140 mg/dL, and maximal glucose level. FMD showed similar trend; greater increase was observed in patients with more significant HbA_1_c improvement. Therefore, our study demonstrated that responders to RT-CGM benefit most in endothelial function improvement. Surprisingly, we observed the greatest increase in FMD in those with optimal glycaemic control from the onset. While HbA_1_c in this subgroup decreased even more from 7.25% to 6.7%, changes in glycaemic variability parameters did not achieve statistical significance, except for the already abovementioned decrease in AUC for hypoglycaemia. We can speculate that it is due to their already initial high adaptation to diabetes-related tasks and educational potential and abilities to view and use real-time data to make insulin, nutrition, and lifestyle modifications. However, the mechanism is still unknown and requires further research. Our data provide important information on the role glycaemic variability may play in influencing cardiovascular risk even in children with T1DM and the possibility to undertake deliberate action to improve endothelial function in this group of high-risk patients.

### 4.1. Limitations of the Study

We are aware that there are certain limitations of our study implicating a careful interpretation of the conclusions. Metabolic control and endothelial function improvement might be caused by extra training and more intensive education schedule that was given to the patients and their caregivers rather than the effect of CGM itself. No control group went through the same training and monitoring without RT-CGM. An additional limitation is that the improvement of A_1_c after 3 months was directly attributed to the 4 weeks of CGM use; there is no proof from the data that these two are directly related. The study was not controlled for physical activity. Our group numbers were quite small, with only 8 patients in the <7.5% group. Notwithstanding, the aim of the study was rather to demonstrate the additional benefits of RT-CGM device use in children and adolescents than to prove the superiority of CGM over SMBG in improving metabolic control and endothelial function.

## 5. Conclusions

RT-CGM can be considered as an additional, educational tool which offers type 1 diabetic adolescent the quick reaction to decrease glycaemic variability parameters in short-time observation. Whether such approach may allow for the improvement in endothelial function and further influence to reduce the risk of future cardiovascular disease remains to be elucidated. The best outcome was demonstrated in initially well-controlled patients (HbA_1_c <7.5%), but also in RT-CGM responders, that significantly improved HbA_1_c and glycaemic fluctuations in one-month system use. Significant FMD improvement was not evident in poorly controlled individuals.

## Figures and Tables

**Table 1:** Basic characteristic of the study group.

	Study group	HbA_1_c before the study		Improved HbA_1_c after 3-month follow-up	
		<7.5%	≥7.5%	Yes	No
Number of patients	40	8	32	27	13
Age (years)	14.6 ± 2.1	15 ± 1	14.5 ± 2	14.4 ± 2	15.1 ± 1
Gender (boys/girls)	19/21	4/6	15/15	15/12	4/9
Diabetes duration (yrs)	7.4 ± 3.6	6.5 ± 2.7	7.6 ± 3.8	7 ± 4	8 ± 2.2
Height (m)	1.6 ± 0.1	1.7 ± 0.1	1.6 ± 0.1	1.6 ± 0.1	1.7 ± 0.0
Body mass (kg)	59 ± 9.9	64 ± 3.8	58 ± 10	58 ± 10	62 ± 8
Body mass index (kg/m^2^)	21.6 ± 3.1	21 ± 1.8	21.8 ± 3	22 ± 3	21 ± 2.4
SDS-BMI	0.6 ± 1.0	0.3 ± 0.8	0.7 ± 1	0.8 ± 1	0.2 ± 0.8
Cholesterol (mg/dL)	167.2 ± 29.1	138.3 ± 9.8	174.4 ± 27.9	169.9 ± 34.4	161.5 ± 11.6
LDL (mg/dL)	88.0 ± 25.7	73.5 ± 10.1	91.7 ± 27.2	88.4 ± 28.6	87.3 ± 19.1
HDL (mg/dL)	64.7 ± 16.7	48.0 ± 3.6	68.8 ± 16.0	67.2 ± 16.7	59.4 ± 15.9
TG (mg/dL)	70.7 ± 21.1	80.8 ± 26.9	68.1 ± 19.1	69.8 ± 16.7	72.4 ± 16.7
SBP (mmHg)	116.1 ± 7.1	125.0 ± 6.5	113.8 ± 5.2	116.6 ± 8.0	114.9 ± 4.7
DBP (mmHg)	71.1 ± 4.9	74.0 ± 5.5	70.3 ± 4.6	71.8 ± 4.8	69.5 ± 5.1
HbA_1_c (%) before study	9.3 ± 1.5	7.2 ± 0.2	9.8 ± 1.2	9.0 ± 1.3	10 ± 1.7

Data are presented as mean ± SD.

**Table 2:** Glucose variability during the study in the whole group and depending on HbA_1_c improvement.

		Study group	HbA_1_c improvement	No HbA_1_c improvement
HbA_1_c (%)	Before the study After the study *p*	9.35 ± 1.5 8.81 ± 1.8 <0.001	9.03 ± 1.35 8.04 ± 1.33^*∗*^ <0.001	10.03 ± 1.71 10.42 ± 1.60^*∗*^ 0.102

Mean glucose (mg/dL)	1st week Last week *p*	168.18 ± 33.9 144.8 ± 22.9 <0.001	164.44 ± 37 138.22 ± 31^*∗*^ <0.001	175.92 ± 22.77 158.46 ± 21.19^*∗*^ <0.001

Mean glucose SD (mg/dL)	1st week Last week *p*	61.25 ± 14.5 53.55 ± 14.1 0.002	60.74 ± 15 51.67 ± 14 0.010	62.31 ± 12.43 57.46 ± 21.19 0.050

AUC > 140 mg/dL	1st week Last week *p*	43.29 ± 26.1 25.23 ± 16.9 <0.001	41.23 ± 29 21.22 ± 15^*∗*^ <0.001	47.56 ± 18.47 33.58 ± 17.41^*∗*^ <0.001

AUC < 70 mg/dL	1st week Last week *p*	0.58 ± 0.61 0.53 ± 0.53 0.591	0.73 ± 0.6^*∗*^ 0.62 ± 0.5 0.439	0.29 ± 0.36^*∗*^ 0.35 ± 0.36 0.576

Max glucose (mg/dL)	1st week Last week *p*	365.15 ± 74.5 328.6 ± 67.2 0.002	344.37 ± 57 317.41 ± 55 0.004	380.6 ± 100.08 354.92 ± 82.98 <0.001

Min glucose (mg/dL)	1st week Last week *p*	51.15 ± 12.5 47.5 ± 6.4 0.045	49.85 ± 13 46.07 ± 5^*∗*^ 0.136	53.85 ± 10.05 50.46 ± 7.31^*∗*^ 0.107

FMD (%)	Before the study After the study *p*	10.9 ± 6.6 16.67 ± 8.5 <0.001	12.22 ± 5.41 19.27 ± 7.40^*∗*^ <0.001	8.18 ± 8.15 11.29 ± 8.35^*∗*^ 0.089

Results of RT-CGM are presented as mean ± SD.

^**∗**^ *p* < 0.05: significant difference between two subgroups, considering the same time sensor usage (first or last week).

**Table 3:** Glucose variability depending on the initial HbA1c level.

		HbA_1_c before the study	
		<7.5%	≥7.5%
HbA_1_c (%)	Before the study After the study *p*	7.25 ± 0.19^*∗*^ 6.73 ± 0.47^*∗*^ 0.006	9.88 ± 1.22^*∗*^ 9.33 ± 1.62^*∗*^ 0.003

Mean glucose (mg/dL)	1st week Last week *p*	127.75 ± 9.84^*∗*^ 126.75 ± 23.26^*∗*^ 0.8	178.28 ± 29.90^*∗*^ 149.31 ± 8.12^*∗*^ <0.001

Mean glucose SD (mg/dL)	1st week Last week *p*	46.75 ± 6.94^*∗*^ 44.75 ± 3.49^*∗*^ 0.286	64.88 ± 13.57^*∗*^ 55.75 ± 14.95^*∗*^ 0.003

AUC > 140 mg/dL	1st week Last week *p*	14.75 ± 6.94^*∗*^ 13.00 ± 3.52^*∗*^ 0.157	50.46 ± 24.33^*∗*^ 28.29 ± 17.65^*∗*^ <0.001

AUC < 70 mg/dL	1st week Last week *p*	0.98 ± 0.46^*∗*^ 0.68 ± 0.28 0.203	0.49 ± 0.61^*∗*^ 0.58 ± 0.62 0.957

Max glucose (mg/dL)	1st week Last week *p*	279.75 ± 25.93^*∗*^ 291.50 ± 14.92 0.14	375.26 ± 70.36^*∗*^ 339.13 ± 71.90 <0.001

Min glucose (mg/dL)	1st week Last week *p*	42.75 ± 1.75^*∗*^ 48 ± 5.45 0.045	52.25 ± 13.17^*∗*^ 47.37 ± 6.65 0.005

FMD (%)	Before the study After the study *p*	16.25 ± 4.32^*∗*^ 27.81 ± 6.49^*∗*^ 0.002	9.57 ± 6.44^*∗*^ 13.89 ± 6.43^*∗*^ <0.001

Results of RT-CGM are presented as mean ± SD.

^**∗**^ *p* < 0.05: significant difference between two subgroups, considering the same time sensor usage (first or last week).
